# Temporal metabolomic fingerprinting identifies adenine as a novel biomarker for early detection of *Escherichia coli* infection in broiler chickens

**DOI:** 10.1038/s41598-025-16873-x

**Published:** 2025-08-27

**Authors:** Asha Ranaraja, Iresha Subhasinghe, Shaik Noor Ahmad, Babajan Banaganapalli, Shelly Popowich, Snijesh V. Parambath, Lisanework E. Ayalew, Rupasri Mandal, David S. Wishart, Suresh Tikoo, Susantha Gomis

**Affiliations:** 1 Department of Veterinary Pathology, Western College of Veterinary Medicine, University of Saskatchewan, 52 Campus Drive, SK S7N 5B4 Saskatoon, Canada; 2 Department of Genetic Medicine, Faculty of Medicine, King Abdulaziz University, Jeddah, Saudi Arabia; 3 Division of Molecular Medicine, St. John’s Research Institute, Bangalore, India; 4 Department of Pathology and Microbiology, Atlantic Veterinary College, University of Prince Edward Island, 550 University Ave, Charlottetown, PE C1A 4P3 Canada; 5 Departments of Biological Sciences and Computing Science, University of Alberta, Edmonton, AB T6G 2E9 Canada; 6 Vaccinology and Immunotherapy, School of Public Health, University of Saskatchewan, Saskatoon, 7 N 5E3 Canada

**Keywords:** Adenine, Broiler chicken, *Escherichia coli*, Metabolic biomarker, Nucleotide metabolism, Bacterial pathogenesis, Animal physiology, Metabolomics, Infectious diseases, Bacterial infection

## Abstract

Avian pathogenic *Escherichia coli* causes septicemia in broiler chickens leading to high mortality and economic losses. Current diagnostic methods, such as serology and culture, cannot detect infections during early asymptomatic stages. Hence, this study focused on identifying novel serum metabolic biomarkers and pathways as an early detection prediction tool. Ross broiler chicks were challenged with *E. coli* at 3 or 5 d of age, and blood samples collected at 8 and 24 h following infection. Serum samples were analyzed for metabolite alterations using targeted The Metabolomics Innovation Centre (TMIC) mega metabolomics assay. Data was processed through comprehensive statistical analyses, including univariate, multivariate, and meta-analysis approaches. At 8 h post-infection, top metabolites like adenine, N-acetyl-alanine, N-acetyl-soleucine, N-acetyl-valine, and orotic acid related to nucleotide and amino acid metabolisms were downregulated (*p* = < 0.05). At 24 h, a distinct metabolic shift emerged with hippuric acid increasing, while adenine showed further depletion, accompanied by decreases in N1-acetylspermidine, N-acetylputrescine, and a modest increase in picolinic acid related to nucleotide, polyamine and immune response pathways (*p* = < 0.05). Correlation metabolite networks show that at 8 h post-infection, broiler chicken showed enhanced metabolic coordination, while at 24 h, disruptions in polyamine, nucleoside, and fatty acid pathways reflected systemic rewiring. The progressive depletion of adenine at both 8 and 24 h post-infection supports it as a novel metabolite signature for *E. coli* infection.

**Supplementary Information:**

The online version contains supplementary material available at 10.1038/s41598-025-16873-x.

## Introduction

The commercial broiler chicken industry is searching for novel measures to combat infections to ensure animal welfare and food safety^1^. High mortality associated with bacterial infections during the first week of a broilers life has devastating impacts on production^2^. Of these bacterial infections, *Escherichia coli* septicemia is a major cause of first-week mortality in the broiler chicken industry worldwide^3^. It has been reported that bacterial infections, primarily *E. coli*, accounted for ∼50% of flock mortalities during the first week^2,4^. In addition to high mortality during the flock cycle, these bacterial infections result in a lack of flock uniformity, chronic infections, and estimated 36–43% of broiler carcasses condemned at processing with lesions consistent with *E. coli* septicemia^3,5^. The high incidence of infectious diseases in neonatal broilers is largely due to immune system immaturity, marked by reduced cytokine production (IL-1β, IL-4, IL-10, IFN-γ), low IL-2 expression in the spleen, and decreased IL-4, IFN-γ, and Lysozyme C expression in the ileum between days 6 and 13^6-8,9^.

To prevent losses due to bacterial infections, antibiotics have been used prophylactically in some poultry sectors; however, increasing regulations now restrict this practice due to concerns over antimicrobial resistance and residues in poultry products^9,10^. Therefore, the use of antibiotics for preventive purposes has been increasingly restricted in many countries. In response to growing concerns about antimicrobial resistance (AMR) and public health, numerous regulatory bodies like the European Union and Food and Drug Administration have implemented stringent policies to limit or ban the use of antibiotics for growth promotion and prophylaxis in livestock^11^.

Alternatives to antibiotics and methodologies for detecting early pathogenic infections are critical in controlling imminent disease outbreaks and reducing antimicrobial use (AMU) to prevent the development of antimicrobial resistance (AMR). There are growing concerns about AMU in poultry production, leading to the emergence of superbugs (bacteria resistant to multiple antibiotics) adversely affecting human, animal, and environmental health^12-14^. One of the strategic priorities of the broiler chicken industry is enhancing consumer trust in chicken meat by managing pathogens and AMU. The early detection of pathogenic infections is critical in controlling pathogens and thus preventing imminent disease outbreaks and AMU.

The broiler chicken industry mostly relies on serological blood testing to measure antibodies against pathogens to detect pathogenic infection. However, serological tests detect diseases only 10–14 days after pathogenic exposure. Besides, polymerase chain reaction (PCR) (pathogen’s DNA detection) and bacterial culture-based diagnosis methods are primarily contingent on the types of tissue and pathogen’s predilection site. The broiler chicken industry lacks the ability to detect pathogens within 1–2 days of post-infection. In this context, metabolomics, the study of small metabolites, is an emerging approach that can identify the early metabolite alterations associated with bacterial infections. In human medicine, the utility of metabolomics for diagnosis of acute diseases is progressing; detection of metabolites in human bio fluids are attractive biomarkers for the diagnosis of early Lyme disease (ELD), a vector-borne infectious disease. Urine represents an easily obtained clinical sample that can be applied for diagnosis of ELD^15^. In contrast, the utility of disease diagnosis utilizing metabolomics in animal industry is in its infancy. Thus, metabolomics approach in diseases diagnosis in the poultry industry will aid in disease prevention and improving the disease management practices.

We hypothesis that metabolic biomarkers are a potential early diagnostic tool associated with *E. coli* septicemia before the onset of clinical signs within 8–24 h (h) post-infection. Hence, the objective of this study was to explore the metabolomics landscape of neonatal broiler chickens following *E. coli* septicemia to identify potential biomarkers in serum associated with acute *E. coli* septicemia. Thus, metabolomics as a tool for disease diagnosis in the field will improve disease management and appropriate timely interventions to reduce losses and to minimize spread of infectious diseases in the poultry industry.

## RESULTS

### Clinical outcome at 8 h Post-*E. Coli* infection

**Fig. 1:**
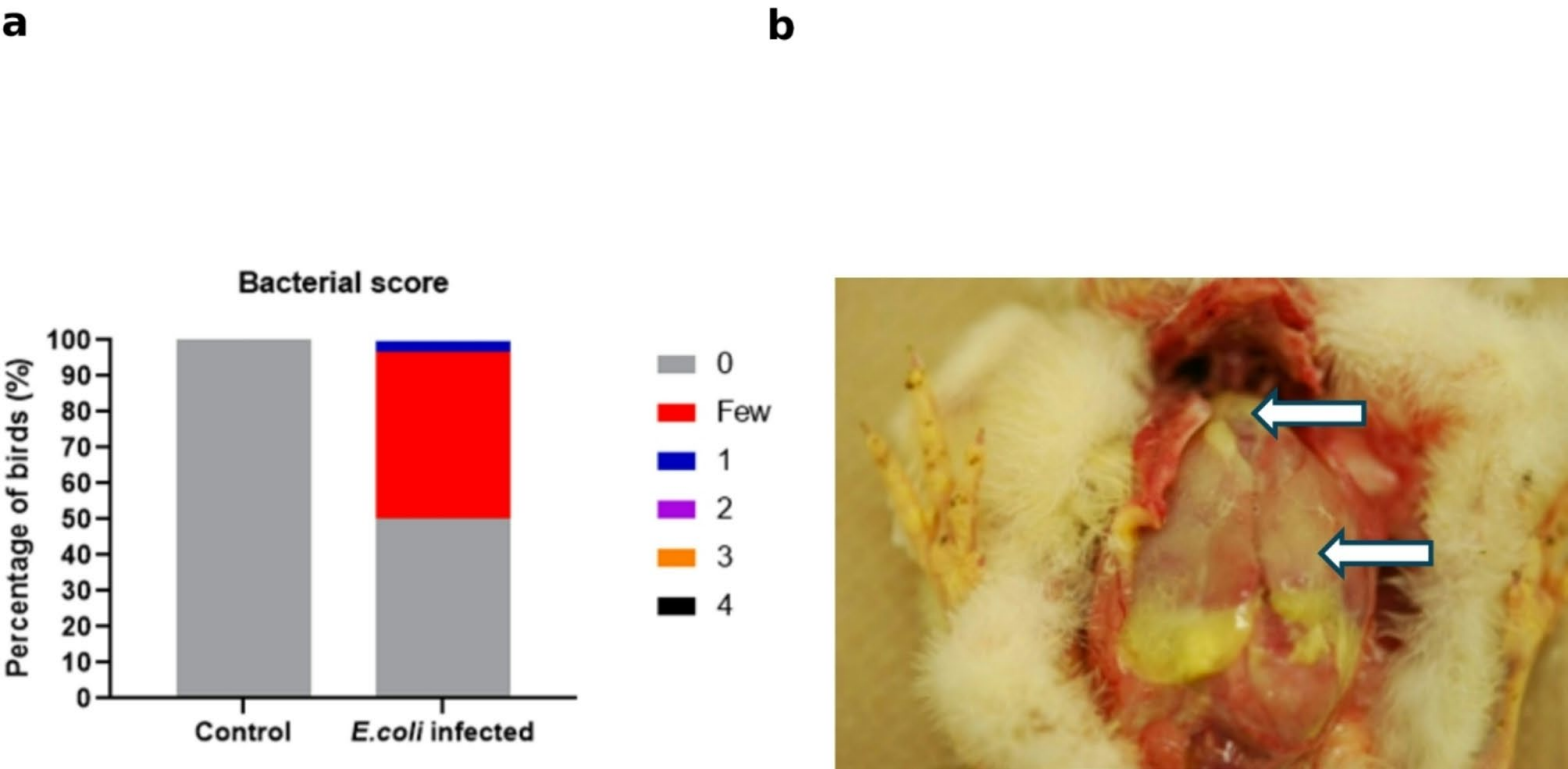
Clinical outcome following ***E. coli*** infection.** a**: This graph illustrates the percentage of birds with different bacterial score ranging from in MacConkey agar after 8 h post*-E. coli* infection. There was no bacterial growth on MacConkey agar from the control birds. The majority of *E. coli* infected birds had “few” *E. coli* growth (46.7%) while a small percentage showed a score of 1 (3.3%). **b**: On necropsy, 24 h post- *E. coli* challenge, the dead bird had fibrin on the heart (pericarditis) and liver (perihepatitis). There is extensive inflammation and fibrinous exudate around the thoracic cavity, and the yellowish discoloration is an indication of fibrin deposition that is causing pericarditis or perihepatitis (white arrows). The surrounding tissues look hyperemic, and the organs are enlarged, indicative of systemic involvement.

All the birds were clinically healthy at 8 h following the *E. coli* challenge. Of the 30 birds challenged with *E. coli*, 15 (50%) yielded bacterial growth from air sac swabs on MacConkey agar, and 10 (33.3%) yielded growth from blood cultures (Fig. 1a**)**. Few birds that were positive for growth on McConkey agar for air sac swabs were found negative for the blood culture and vice versa. However, 66.7% of the birds were positive for either of these tests. Further, the *E. coli* counts in the blood culture ranged from 1 × 10^2^ colony forming units (CFU)/mL to 3 × 10^2^ CFU/mL. This finding suggests that the bacteria likely disseminated systemically in the challenged birds as early as 8 h post-infection, evidenced by their presence in either blood or air sac samples. No clinical signs or mortality were observed in the group inoculated with saline. Bacterial growth on culture plates were scored as follows: 0 = no bacterial growth, few = 1–5 colonies, 1 = bacterial growth in the first quadrant, 2 = bacterial growth in the first and second quadrants, 3 = bacterial growth in the first, second, and third quadrant, and 4 = bacterial growth in all four quadrants. Among the 30 infected birds tested for bacterial load in air sacs, the majority exhibited “few” bacterial growths in McConkey agar (46.7%), with 1 bird (3.3%) scoring 1. No *E. coli* growth was observed on MacConkey agar from the rest of the birds (50%). These results indicate that there is variability in the colonization of *E. coli* in the infected birds.

### Clinical outcome at 24 h Post-*E. Coli* infection

At 24 h post-infection, one bird from the challenged group died (3.3% mortality) while the rest of the birds appeared healthy without any clinical signs. On necropsy, the dead bird had polyserositis (combination of airsacculitis, pericarditis and perihepatitis) (Fig. 1b**).**

### 8 h metabolite response to *E. coli*

**Fig. 2:**
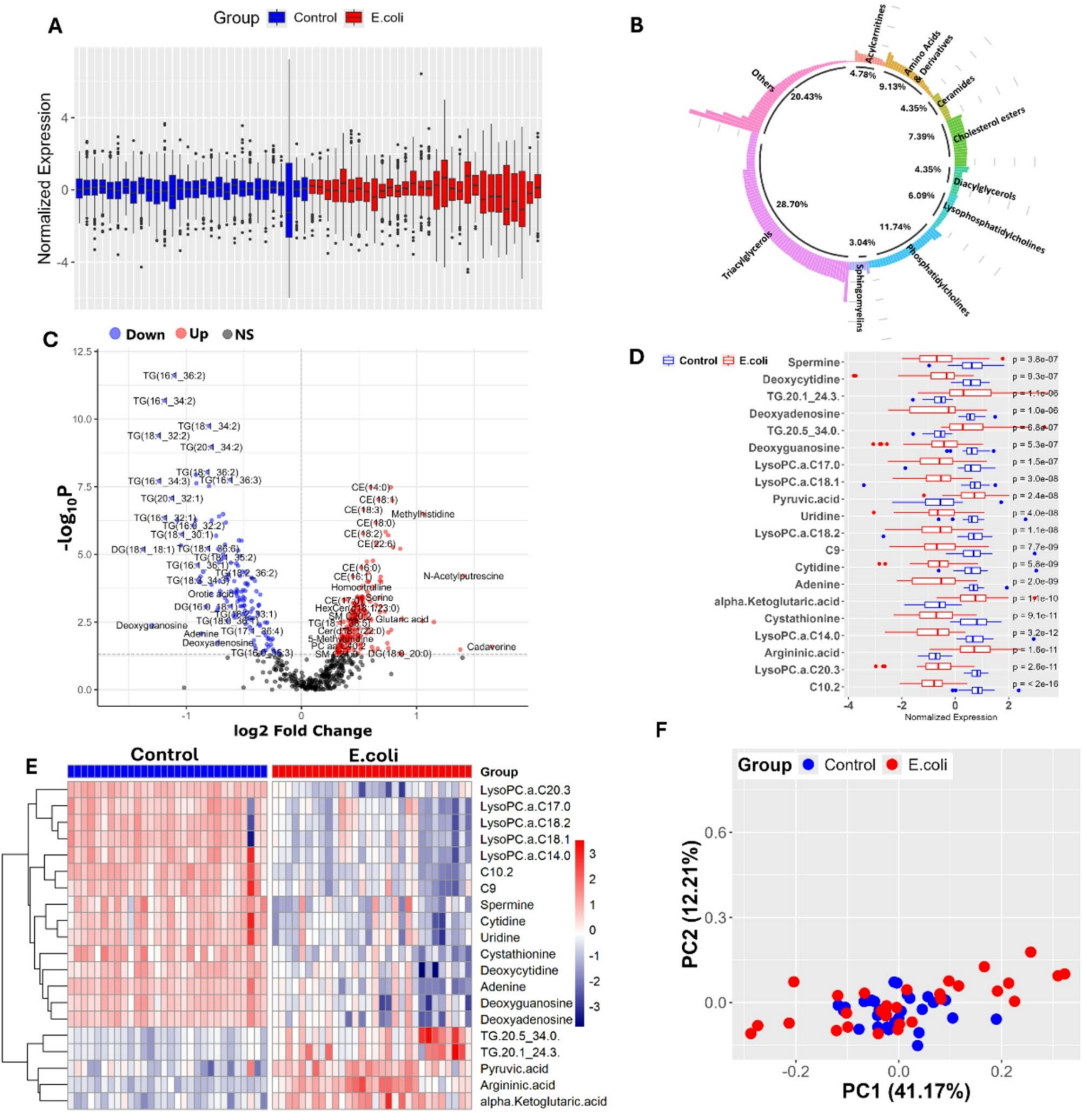
Metabolomics analysis of the ***E. coli*** (8 h) infected group vs. the control group.** (a)** Boxplot of normalized metabolite expression for control (blue) and *E. coli*-infected (red) samples. **(b)** Circular plot of metabolite class distribution. **(c)** Volcano plot displaying differentially expressed metabolites. **(d)** Boxplots comparing normalized expression of selected metabolites in both groups. **(e)** Heatmap of the top 20 differentially expressed metabolites between groups. **(f)** PCA plot showing separation between control and infected groups.

This metabolomics data underwent preprocessing to ensure consistency and data quality. In brief, the data was standardized by removal of constant features, imputation of low limit of detection (LOD) values with half minimum positive value, median scaling by log (base 10) transformation and mean centering (Fig. 2a) (Table S1A). Of the total 598 metabolites, univariate analysis has identified 335 (56.02%) differentially expressed metabolites in *E. coli* infected birds compared to control birds (*p = <* 0.05). These 335 metabolites belong to several classes including triacylglycerols (40%), phosphatidylcholines (17.01%), acylcarnitines (5.67%), amino acids and derivatives (5.37%), ceramides (4.78%), cholesterol esters (4.18%), diacylglycerols (3.58%), lysophosphatidylcholines (3.37%), and sphingomyelins (1.19%) and others (15.52%) (Fig. 2b). Of these 335 differentially expressed metabolites, 134 (40%) were down regulated [log2 fold change (Log2FC)] range was − 1.37 to −0.88; *p = <* 0.05) and 201 were up regulated (Log2FC range is 0.30 to 4.56; *p = <* 0.05) as presented in the volcano plot (Fig. 2c) (Table S1B). The metabolite concentrations and expression patterns of the top 20 differentially expressed metabolites in *E. coli* and control chicken are shown in the form of box plots (Fig. 2d**)** and heatmaps (Fig. 2e).

The principal component analysis (PCA) plot separated the *E. coli* infected group (in blue) and control group (in red) (Fig. 2f). Principal component (PC)1 was responsible for 48.69% and PC2 for 9.48%. Both components show the variance in the metabolite profile between the two groups. Although there is some overlap between PC1 and PC2, distinct clustering of metabolic differences due to *E. coli* infection is observed. This implies that *E. coli* infection has a noticeable impact on the serum metabolite composition in broilers.

**Fig. 3:**
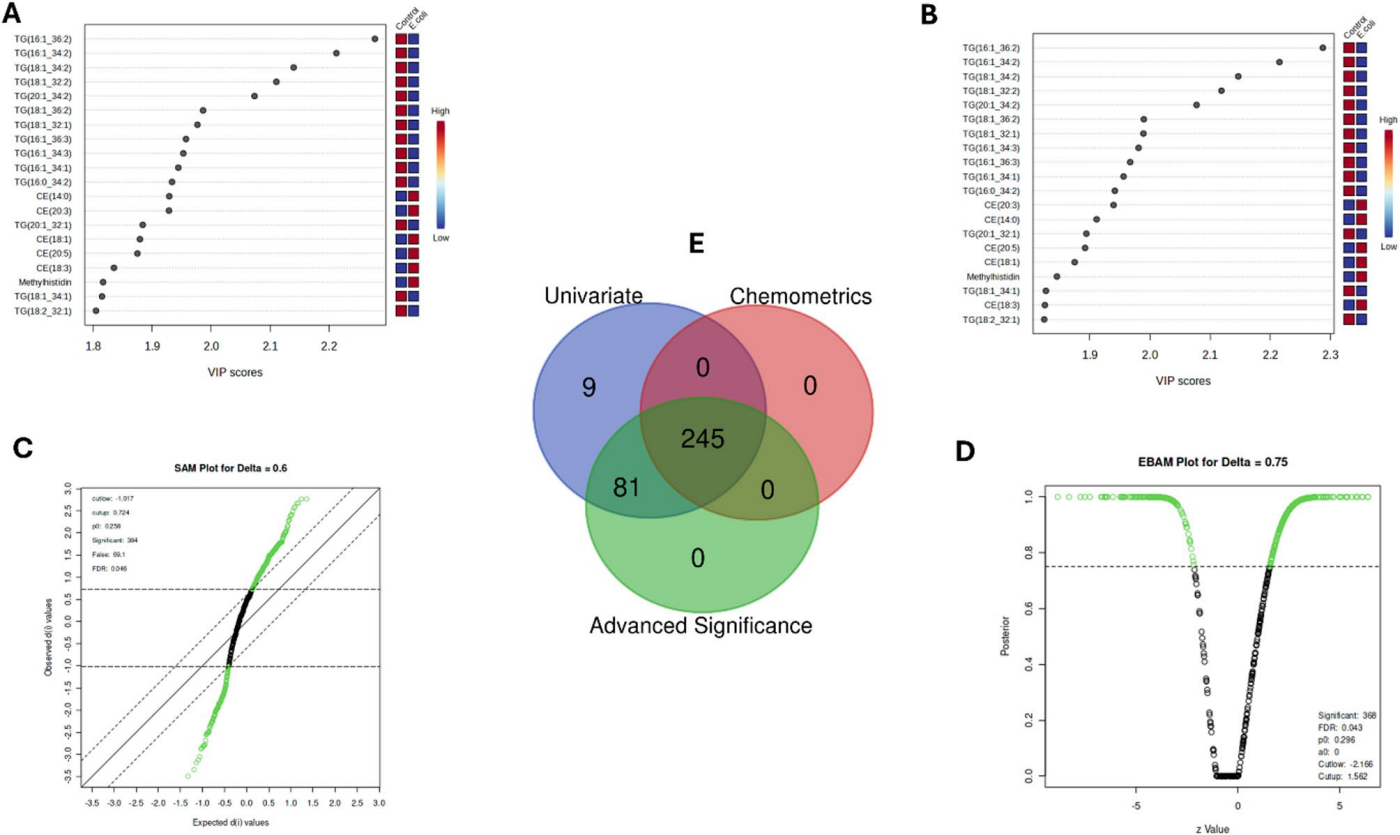
Comprehensive chemometric and statistical profiling of metabolomic alterations post ***E. coli*** challenge (8 h) in broiler chickens.** (a)** PLS-DA VIP scores for key discriminant metabolites distinguishing between the *E. coli*-infected and control groups. **(b)** OPLS-DA VIP scores confirmed PLS-DA findings with enhanced class separation. **(c)** SAM plot showing significant metabolites with differential abundance (delta = 0.8). **(d)** EBAM plot representing significant metabolites with posterior probabilities. **(e)** Venn diagram showing overlaps between univariate, chemometric, and advanced significance analyses, with 245 metabolites consistently identified by all methods.

In a partial least squares discriminant analysis (PLS-DA) model, a variable importance in projection (VIP) score plot demonstrates the highest discriminatory power of metabolites in distinguishing *E. coli* infected and control groups based on their expression values (Fig. 3a) (Table S1C)**.** We found that 242/594 (40.74%) of metabolites had > 1 VIP score. The top VIP scoring over expressed metabolites includes triglyceride (TG) (16:1_36:2) with a score of 2.27, TG (16:1_34:2) with a score of 2.21, and TG (18:1_34:2) with a score of 2.13. Conversely, among the lowest VIP scoring metabolites showing reduced expression within the *E. coli*-infected group were methylhistidine (VIP score − 1.8167), TG (18:1_34:1) (VIP score − 1.81), and TG (18:2_32:1) (VIP score − 1.80). The result of the orthogonal partial lease squares-discriminant analysis (OPLS-DA) also further supported the similar trend of metabolite discrimination, as shown in Fig. 3b (Table S1D). Among the 593 metabolites analyzed, 243 (40.97%) of metabolites had a VIP score greater than 1. In particular, the OPLS-DA VIP scores of the top three overexpressed metabolites identified in the PLS-DA model showed consistency: TG (16:1_36:2), with a VIP score of 2.28; TG (16:1_34:2), with a VIP score of 2.21; and TG (18:1_34:2), with a VIP score of 2.14. The concordance in the findings of PLS-DA and OPLS-DA underlines the importance of TG metabolism as a robust biomarker of changed metabolic responses to *E. coli* infection and further emphasizes that lipid metabolism plays an important role in the host’s defense against infection.

### Metabolic shifts identified by SAM and EBAM at 8 h

By the SAM analysis, 326 (54.51%) of the 598 queried metabolites were statistically significant. Among these, 193 (59.50%) were up-regulated, while 132 (40.49%) were down-regulated according to the d-value scores of the up- and downregulated metabolites in the range from 1.02 to 2.76 and from − 3.49 to −1.02, respectively, with a *p* = < 0.05 (Fig. 3c) (Table S1E). The highest upregulated metabolite, CE (14:0) (d = 2.77 and *p* = < 0.05), has a role in cholesterol-ester metabolism, and may likely modulate immune response to infection with *E. coli*. Methylhistidine, another upregulated metabolite, had a d-value of 2.56 and a *p* = < 0.05, indicating an increase in muscle protein turnover and metabolic response related to stress. Meanwhile, butyric acid and isobutyric acid were also increased, with a d-value of 2.27 (*p* = < 0.05), indicating an increased generation of short-chain fatty acids in the adaptive process to infection-induced stress. On the other hand, TG (16:1_36:2) exhibited downregulation, with a d-value of −3.49 (*p* = < 0.05), indicating a reduction in the levels of TG due to infection and also pointing toward disturbances in lipid metabolism. The additional downregulated metabolite is TG (18:1_34:2) (d-=−3.18 and *p* = < 0.05) reflects changes in triglyceride metabolism. Furthermore, TG (20:1_34:2), is also decreased (d=−3.05, and *p* = < 0.05), underlines further disturbances in energy storage and mobilization following exposure to *E. coli*.

Subsequently, EBAM analysis showed that of 598 metabolites, 268 metabolites (44.82%) were statistically significant (Fig. 3d) (Table S1F). Among these, 113 metabolites were upregulated, whose z-values ranged between − 8.84 and − 2.81 (*p* = < 0.05), while 155 were downregulated, with z-values ranging between 2.47 and 6.37 (*p* = < 0.05). Among all the upregulated metabolites, TG (16:1_36:2) was elevated the most, indicating disturbance of lipid metabolism, while among the downregulated metabolites, the lowest level observed was for CE (14:0), suggesting marked disturbances of cholesterol esters metabolism after *E. coli* infection.

The Venn diagram (Fig. 3e) shows 245 overlapping metabolites across the student’s t-test, PLS-DA, OPLS-DA, SAM, and EBAM tests. The intersection thus represents key metabolite biomarkers involved broilers response to *E. coli* infection (Table S1G).

### 24 h metabolite response to *E. coli*

**Fig. 4:**
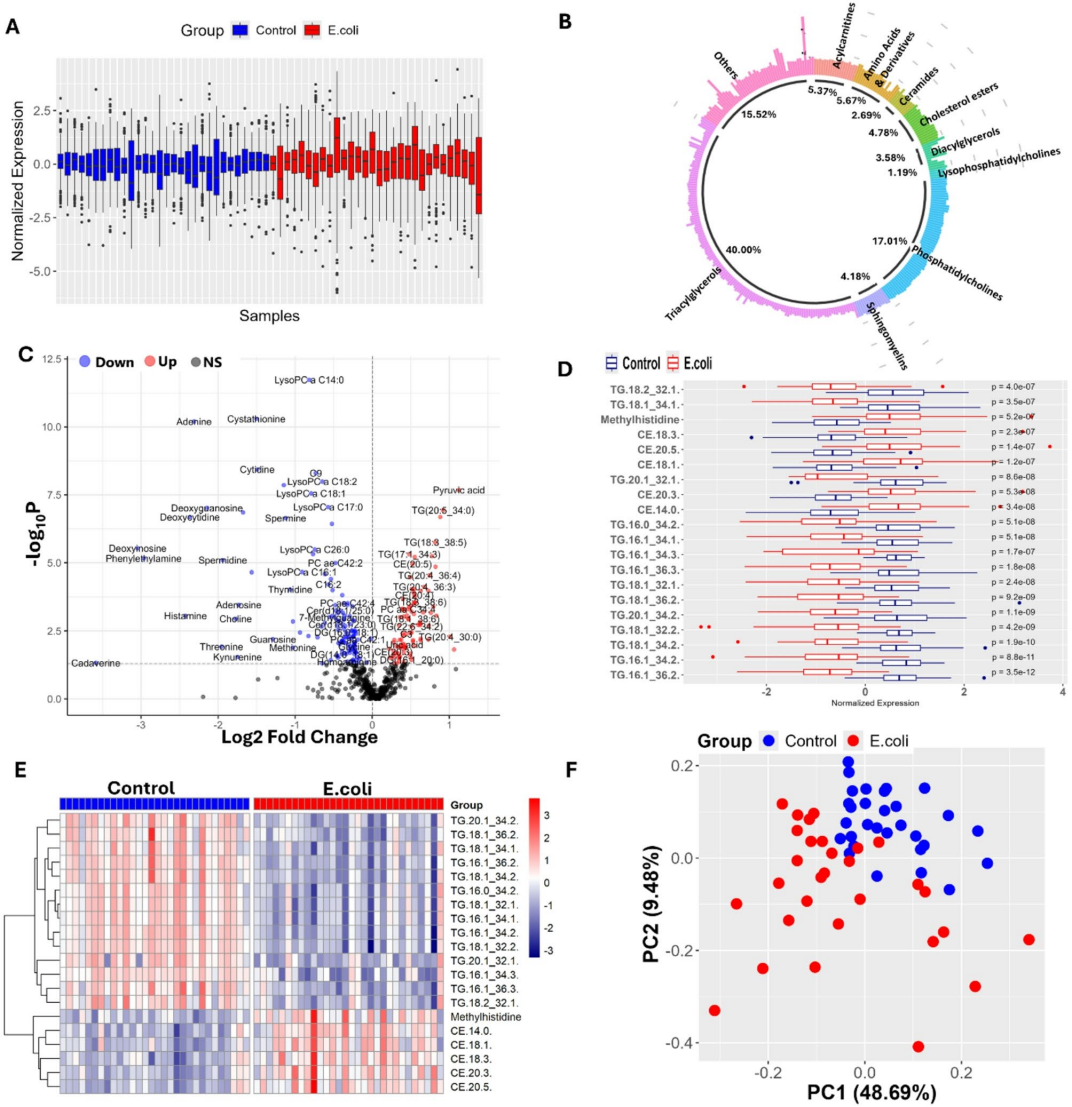
Metabolomics analysis of the ***E. coli*** (24 h) infected group vs. control group.** (a)** Volcano plot showing differentially expressed metabolites. **(b)** Circular plot showing metabolite class distribution. **(c)** Hierarchical clustering of samples based on metabolite profiles. **(d)** Box plots comparing metabolite levels between control and infected groups. **(e)** Heatmap of the top 20 differentially expressed metabolites between groups. **(f)** PCA plot showing separation between control and infected groups.

The raw serum metabolomics data of both infected and control group chickens was preprocessed in order to improve their over quality and structure. This process excluded variables that had more than 80% missing values, imputed values below the analytical limit of detection (1/2 LOD), omitted constant variables and normalized data through median, transformed data using the Box-Cox transformation, log (base 10) and mean-centered based data scaling (Table >S2A). These steps were necessary for reproducibility and harmonization of metabolomics results **(**Fig. 4a**).** Using an univariate analysis, 230 (38.46%) of 598 metabolites were identified to be statistically significant in the *E. coli-*infected group compared to the control group (*p = <* 0.05) (Table S2B). Of these 230 metabolites, 28.70% are triacylglycerols, while 11.7% are phosphatidylcholines, 9.1% are amino acids and derivatives and 7.4% are cholesterol esters. Other important classes are lysophosphatidylcholines, 6.09%; acylcarnitines, 4.78%; ceramides, 4.35%; diacylglycerols, 4.35%; sphingomyelins, 3.04%; and other remaining metabolites, 20.43% **(**Fig. 4b**).** Among all the detected metabolites, 96 metabolites (41.73%) were significantly up-regulated and their log base 2-fold change LogFC ranged between 0.22 and 2.86 and a *p*-value of < 0.05; 134 metabolites (58.26%) were significantly down regulated with LogFC ranging from − 3.58 to −0.01 and a *p-*value of < 0.05, as shown in the volcano plot (Fig. 4c**).** The box plots (Fig. 4d**)** and heatmaps (Fig. 4e**)** represent the metabolite concentrations and expression patterns of the top 20 metabolites in the *E. coli* infected and control groups.

The PCA plot (Fig. 4f) shows the distribution of the *E. coli*-infected and control groups in a two-dimensional space defined by the two PC (PC1 and PC2). PC1 describes 41.7% while PC2 describes 12.17% of the total variance in data. The clustering pattern of the samples reveals some overlap between infected and control groups, which indicates shared metabolic profiles, while highlighting distinct groupings that suggest differential metabolic signatures in the *E. coli*-infected group compared to the control group.

**Fig. 5:**
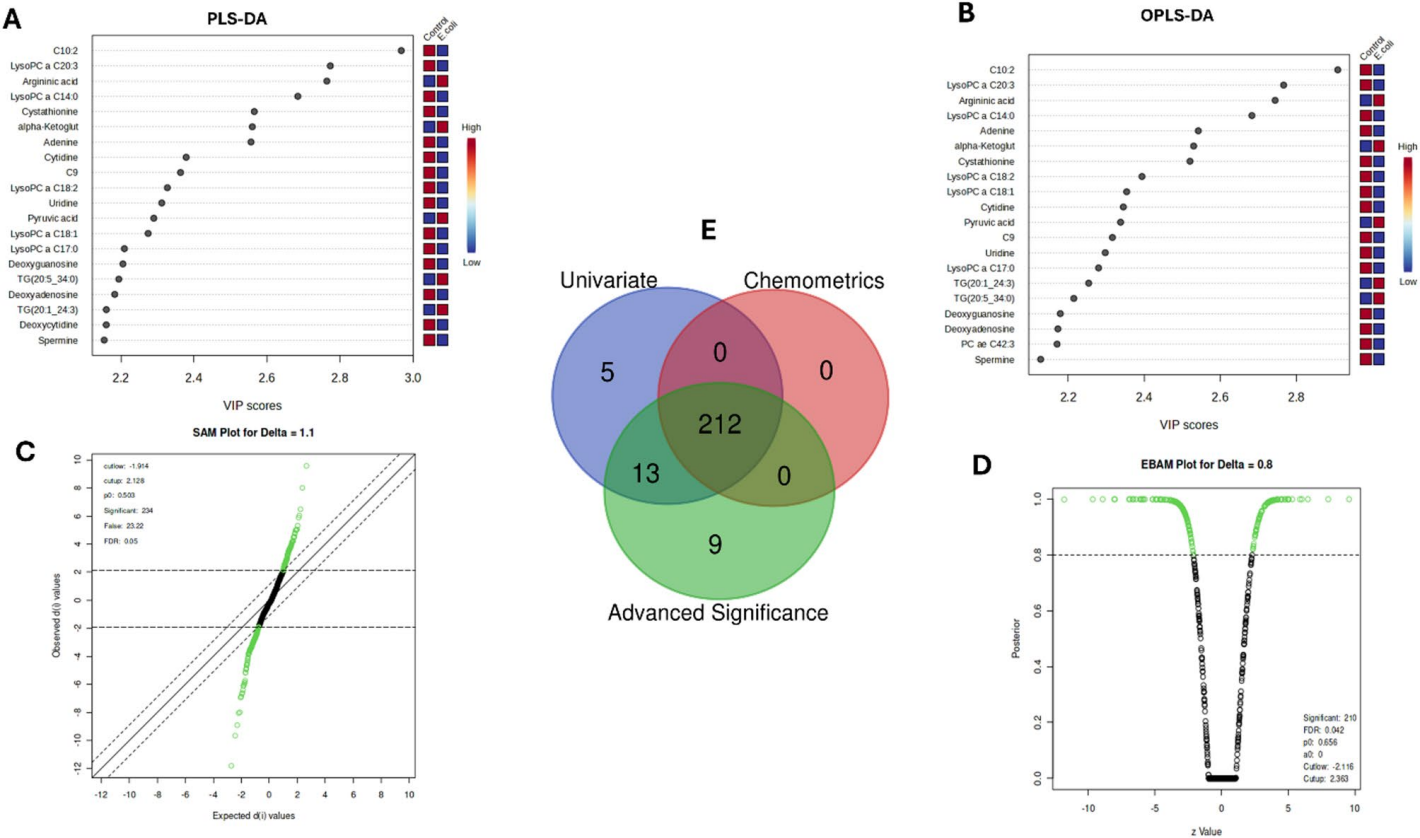
Comprehensive chemometric and statistical profiling of metabolomic alterations post- ***E. coli*** challenge (24 h) in broiler chickens.** (a)** PLS-DA VIP scores for key discriminant metabolites in distinguishing between the *E. coli*-infected vs. control groups. **(b)** The OPLS-DA VIP scores further confirmed the findings of the PLS-DA, with adequate class discrimination. **(c)** SAM plot showing metabolites rated as significant by their differential abundance. **(d)** EBAM plot showing the significant metabolites and their posterior probabilities under *E. coli* infection. **(e)** Venn diagram: overlaps from metabolites identified by univariate, chemometric, and advanced significance analysis showing the consistency of findings.

The VIP score plot indicates the metabolites with the highest discriminant power for the PLS-DA model in distinguishing between *E. coli* infected and the control group (Fig. 5a) (Table S2C). Out of the 599 metabolites analyzed, 206 (34.39%) metabolites possessed VIP score of more than 1. The top overexpressed metabolites included argininic acid (VIP score: 2.75), which improves the immune response through nitric oxide production, alpha-ketoglutarate (VIP score: 2.58), which supports energy production, and pyruvic acid (VIP score: 2.29) that meets increased energy demands during the infection, and TG (TG 20:5, TG 20:1) (VIP scores: 2.2, 1.90), which modulate lipid metabolism and energy storage. On the other hand, metabolites that were downregulated in the *E. coli*-infected group, such as C10:2 (VIP score: 2.96) and LysoPC a C20:3 (VIP score: 2.8), reflect the shift in lipid metabolism pathways due to physiological stress during infection. Further cross-validation with the established model using OPLS-DA affirms these observations of PLS-DA with further enhanced class discrimination and the isolation of the variation that is orthogonal to the VIP scores (Table S2D). Out of 599 metabolites, 207 (34.61%) metabolites had a VIP score of more than 1. The top metabolites identified by OPLS-DA include arginine acid, alpha-ketoglutarate, pyruvic acid, TG (TG 20:TG 16:0–18: 1, TG 18: 0–18: 1, TG 20: 1, C10: 2, and LysoPC a C20:3 (Fig. 5b). These OPLS-DA validate the key metabolites identified by PLS-DA method and reinforce the metabolic changes required for compensating the physiological and immune demands of infection in chicken.

## Metabolic shifts identified by SAM and EBAM at 24 h

By SAM analysis, 234/598 (39.13%) metabolites were determined to be statistically significant (*p* = < 0.05) (Table S2E). Of which, 96 (41.02%) metabolites were upregulated and 138 (58.97%) were downregulated according to the d-value scores with the range of 9.5 to 2.12 and − 11.80 to −1.9 respectively in 598 queried metabolites [false discovery rate (FDR) < 0.05] (Fig. 5c). Among these upregulated metabolites, argininic acid displayed the highest increase (d.value = 9.57, *p* = < 0.05), indicating major alterations in arginine metabolism that are probably related to the physiological stress response due to *E. coli* infection. Furthermore, alpha-ketoglutaric acid showed a very significant increase with a d.value of 8.0145 and a *p* = < 0.05, indicating an increased production of energy through the tricarboxylic acid (TCA) cycle. Pyruvic acid also exhibited significantly enhanced glycolytic activity, likely reflecting its role in rapid energy provision, with a d.value of 6.48 and a *p* = < 0.05. TG (20:5_34:0) showed increased TG mobilization so as to meet the increased energy requirement associated with infection (d.value = 6.04 *p* = < 0.05).

On the contrary, out of the top three most downregulated metabolites, C10:2 had a z-value of −11.807 with a *p* = < 0.05, hence, highly diminished fatty acid metabolism could implicate disturbed lipid mobilization due to *E. coli* infection. Among these, the most significantly downregulated metabolites included LysoPC a C20:3 (z.value = −9.66, *p* = < 0.05) and LysoPC a C14:0 (z.value = −8.91, *p* = < 0.05), demonstrating disturbances in lysophosphatidylcholine metabolism and thus may indicate defective membrane remodeling and decreased phospholipid availability under the infection.

Subsequent to the SAM analysis, EBAM also provided strong evidence for the alteration in the levels of metabolites in the *E. coli* infected group (Fig. 5d) (Table S2F). A total of 210 (35.11%) out 598 metabolites were determined to be statistically significant. These results indicated the overall increased energy metabolism and stress response in the infected birds as evidenced by the up regulation of argininic acid (z-value = 9.576, *p* = < 0.05), alpha-ketoglutaric acid (z-value = 8.01). In contrast, metabolites such as LysoPC a C20:3 (z-value = −9.66, *p* = < 0.05) and LysoPC a C14:0 (z-value = −8.91, *p* = < 0.05) were downregulated implying that the membrane lipid metabolic process and phospholipid synthesis ability are inhibited during infection stress. These changes demonstrate the metabolic shift that occurs during infection.

The Venn diagram shows 212 metabolites that were commonly identified by univariate analyses, such as t-test and chemometric analyses such as PLS-DA and OPLS-DA, and advanced significances using SAM and EBAM, thereby giving strength to these results (Fig. 5e) (Table S2G). This overlap indicates a high degree of similarities in metabolite analysis via different methods. This further consolidates the reliability of those key metabolic alterations in response to *E. coli* infection.

### 8 h vs. 24 h: metabolite and pathway differences

**Fig. 6:**
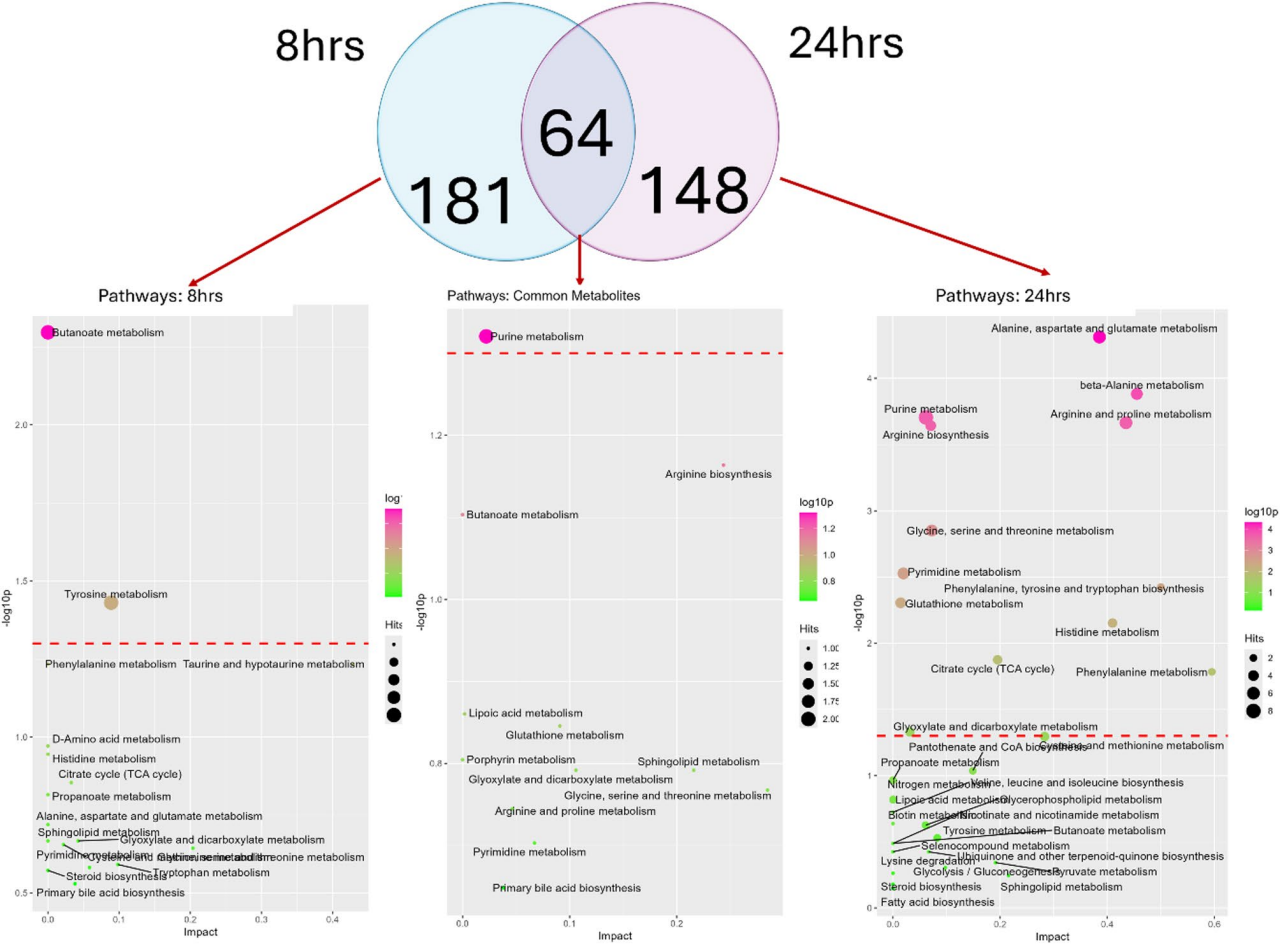
Identification of common and unique metabolites and enriched pathways across 8 h and 24 h post-***E. coli*** challenge: The Venn diagram shows 181 unique metabolites at 8 h, 148 at 24 h, and their overlap (64 common metabolites). Pathway enrichment analysis is shown for the unique as well as common metabolites of 8 and 24 h datasets.

The pathway analysis of 245 metabolites at 8 h post-*E.coli* infection and 212 metabolites from 24 h post-infection data reveals distinct and overlapping metabolic changes **(**Fig. 6). In 8 h post-*E. coli* infection showed that butanoic acid and succinate metabolites were enriched in butanoate metabolism, which is associated with short-chain fatty acid metabolism. Furthermore, tyrosine metabolism was also enriched, involving L-noradrenaline and 4-hydroxyphenylacetate metabolites (*p* = < 0.03) (Table S3A). The list of unique metabolites specific to 24 h infection data consisted of 148 metabolites and showed several significantly enriched pathways. Alanine, aspartate, and glutamate metabolism had the highest significance: *p* = < 4.89E-05, impact = 0.38; metabolites include L-aspartate, L-alanine, L-glutamine, citrate, pyruvate, and 2-oxoglutarate. The following was beta-alanine metabolism: *p* = < 0.0001; key metabolites take part in beta-alanine, L-aspartate, L-histidine, spermidine, and spermine. Other significantly enriched pathways were those of purine metabolism, with *p* = 0.0001, involving metabolites like L-glutamine, adenosine, deoxyadenosine, deoxyinosine, hypoxanthine, guanine, urate, and guanosine; this represents large changes in nucleotide metabolism. Arginine biosynthesis (*p* = < 0.0002) was enriched with metabolites such as L-aspartate, L-ornithine, ammonia, L-glutamine, and 2-oxoglutarate, suggesting involvement in amino acid synthesis. Additional pathways, such as glutathione metabolism (*p* = < 0.004) with L-ornithine, putrescine, spermidine, and spermine, and pyrimidine metabolism (*p* = < 0.002) featuring L-glutamine, cytidine, deoxycytidine, thymidine, and beta-alanine, were also enriched, pointing toward changes in antioxidant defense and nucleotide metabolism. Histidine, phenylalanine, TCA cycle, glyoxylate and dicarboxylate metabolisms were also detected, highlighting shifts in energy metabolism and amino acid biosynthesis specific to batch 2 (*p* = < 0.05) (Table S3B). The enrichment analysis of the 64 common metabolites between 8 h and 24 h, identified purine metabolism as a significantly enriched pathway (*p* = 0.04), involving metabolites like deoxyguanosine and adenine (Table S3C**).**

### Logistic regression (LR) highlights early biomarkers of *E. coli* challenge

**Fig. 7:**
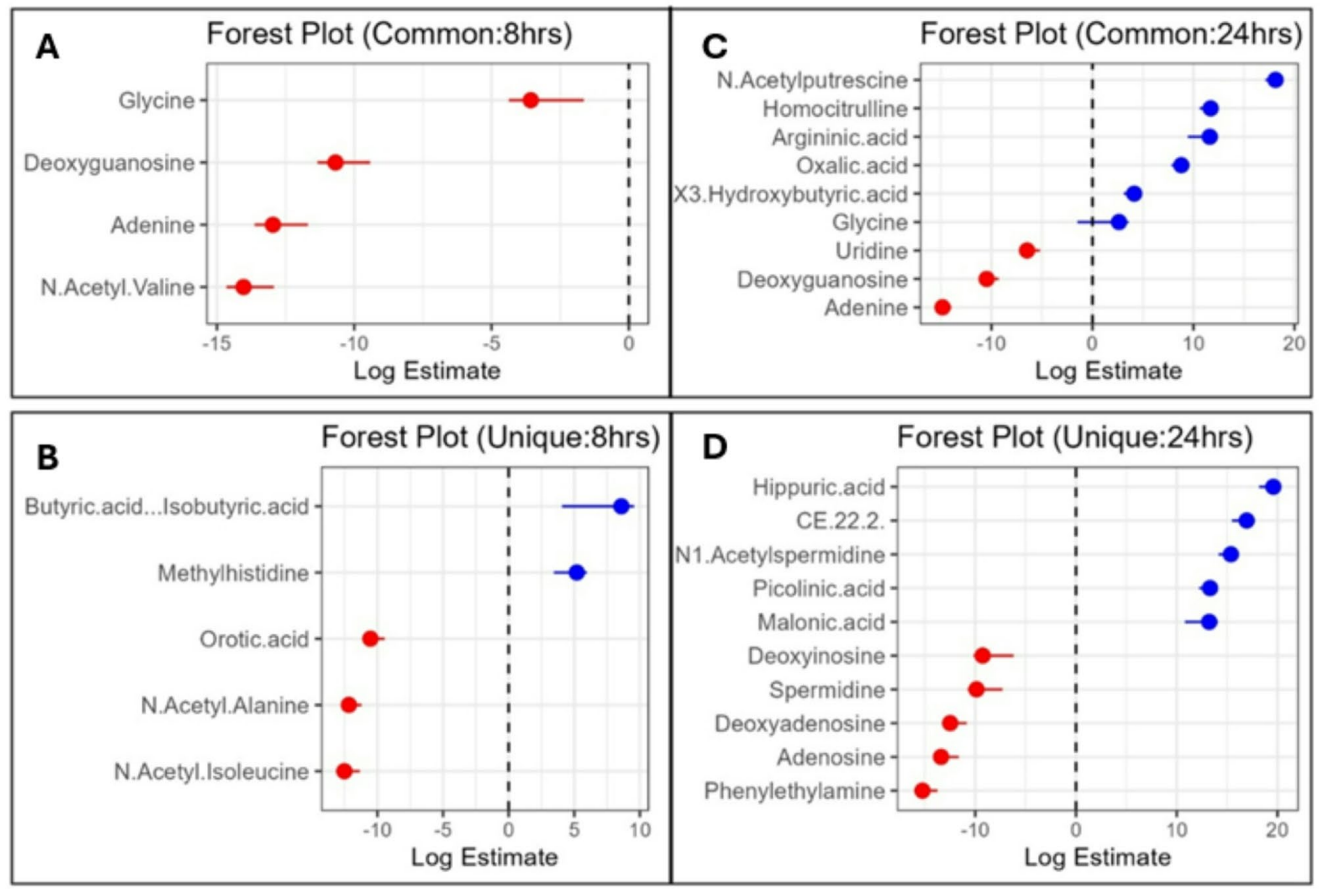
Forest plots of serum metabolite changes in broilers post-***E. coli*** infection at 8 and 24 h. Panel A and B shows unique and common metabolites in 8-h data, whereas panels C and D show unique and common metabolites in 24 h data. Red and blue dots are for negative and positive log odds estimates, respectively, with error bars showing confidence intervals.

LR analysis can predict the infection status by weighing metabolite effect sizes, where odds ratios > 1 imply increased disease risk and < 1 indicate the protective effects, thereby facilitating in disease biomarker identification. The filtering of 393 metabolites between the two batches (8 h and 24 h post-*E. coli* infection) based on the pathway enrichment status identified a total of 91 metabolites, including 24 metabolites from 8 h, 51 metabolites from 24 h to 16 common metabolites (both batches). The LR analysis of these 91 metabolites identified 48 significantly enriched metabolites (*p = <* 0.05). Of these, batch 1 (8 h) had 9 metabolites (5 unique, 4 common), and batch 2 (24 h) had 39 metabolites (30 unique, 9 common) showing the different temporal metabolic signatures of infection progression (Table S3D and S3E) (Fig. 7a-d).

**Fig. 8:**
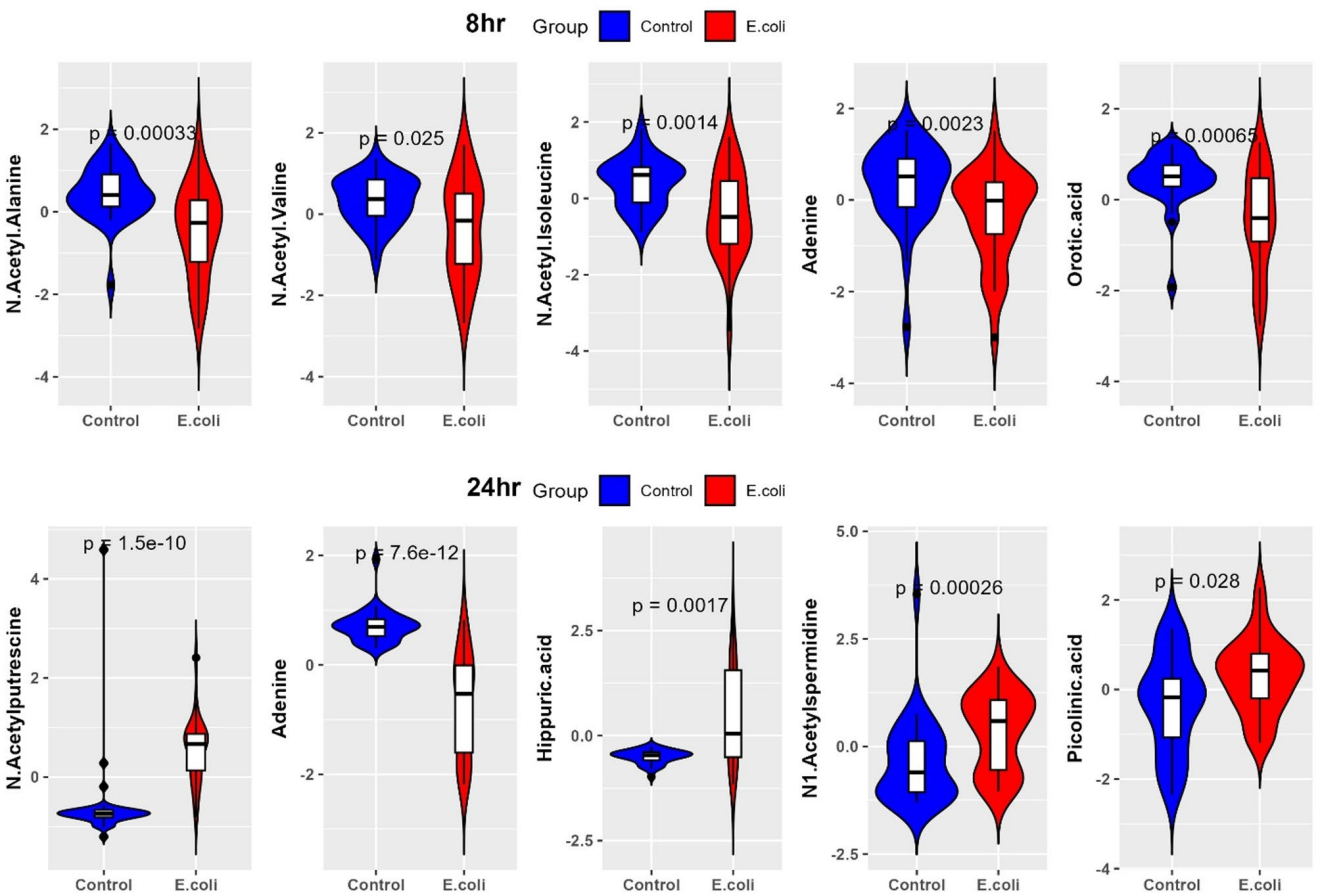
Violin plots showing expression levels of the metabolic biomarker panel in broiler chickens at 8 and 24 h post-***E. coli*** infection. N-acetyl-alanine, N-acetyl-valine, N-acetyl-isoleucine, adenine, and orotic acid were downregulated at 8-h post-infection. N-acetylputrescine, hippuric acid, N1-acetylspermidine, and picolinic acid were upregulated, while adenine remained downregulated at 24 h post-*E. coli* infection.

At 8 h post-*E. coli* challenge, LR results showed significant alterations in metabolites involving amino acid, fatty acid, and nucleotide metabolisms. Butyric acid/isobutyric acid (log estimate: 8.59, *p* = < 0.04) was significantly upregulated, suggesting early infection stage leads to the changes in short chain fatty acid metabolism and gut microbiome composition. Log estimate means for each 1 µM increase in butyric acid/isobutyric acid, the log odds of infection increase by 8.59 units. Methylhistidine (log estimate: 5.20, *p* = < 0.005) also showed positive association and may represent muscle protein catabolism as a response to infection. In contrast, N-acetyl-isoleucine (log estimate: −12.52, *p* = < 0.0004) and N-acetyl-alanine (log estimate: −12.18, *p* = < 6.65E-05) had strong negative associations indicating early dysregulation of amino acid metabolism (Table S3D). The marked depletion of orotic acid (log estimate: −10.53, *p* = < 0.0002) implies the altered nucleotide biosynthesis. However, based on the consensus scores (CS) calculated by combining effect size and statistical significance, the top metabolites were N-acetyl-alanine (CS = 50.86), N-acetyl-valine (CS = 50.34), N-acetyl-isoleucine (CS = 41.86), and orotic acid (CS = 38.66). Additionally, we identified the depletion of deoxyguanosine (CS = 33.25) and glycine (CS = 7.51). Their expression values are presented in the form of violin plots in Fig. 8. These findings suggest early dysregulation of nucleotide biosynthesis and amino acid metabolism as part of the host’s strategy to limit bacterial growth and manage oxidative stress during early *E. coli* infection.

At 24 h post *E. coli* challenge, the metabolites involving amino acid and energy metabolism were significantly altered. Hippuric acid (*p* = < 0.001, log estimate: 19.59, CS = 53.78) was the top-ranked metabolite, indicating increased metabolism of aromatic amino acids and potential alterations in gut microbiota. N1-acetylspermidine (*p* = < 0.0005, log estimate: 15.38, CS = 49.82) and picolinic acid (*p* = < 0.0002, log estimate: 13.31, CS = 48.38) were also ranked high, reflecting disruptions in polyamine metabolism and nicotinamide pathway activity, respectively. Furthermore, CE (22:2) (*p* = < 0.002, log estimate: 16.95, CS = 45.42) revealed profound changes in lipid metabolism, attesting to the metabolic adjustment of the host to infection. Additionally, downregulated metabolites like phenylethylamine, adenosine, deoxyadenosine, spermidine, and spermine indicated major changes in nucleotide, amino acid and stress response pathways (*p* = < 0.05). These findings indicate coordinated metabolic reprogramming during late stages of *E. coli* infection reflecting the host’s adaptation to counteract bacterial growth and manage inflammation.

A comparison of the common metabolites at 8 and 24 h post-infection revealed sustained dysregulation in nucleotide metabolism, with a significant downregulation of deoxyguanosine (8 h: log estimate: −10.68, *p* = < 0.0007; 24 h: log estimate: −10.47, *p* = < 0.0005) and adenine (8 h: log estimate: −12.96, *p* = < 0.0008; 24 h: log estimate: −14.84, *p* = < 1.40E-05). Glycine showed a significant shift from a negative association at 8 h (log estimate: −3.57, *p* = < 0.007) to a positive association at 24 h (2.63, *p* = < 0.03), suggesting temporal shifts in amino acid metabolism. At 24 h, some common metabolites such as N-acetylputrescine (log estimate: 18.12, *p* = < 9.49E-05), homocitrulline (log estimate: 11.70, *p* = < 0.0001), and argininic acid (log estimate: 11.61, *p* = < 0.01) were altered. Their expression values are presented in the form of violin plots in Fig. 8. These changes indicated progressive changes in the urea cycle and polyamine metabolism with the infection time course. While prominent at 24 h, these metabolites were not significantly altered at 8 h as per LR findings.

## Differential correlation patterns in metabolites at 8 h and 24 h

Correlation analysis uses coefficient of determination (R²) scores, where higher values indicate stronger correlations between metabolite pairs. A ΔR² score derived from correlation difference control and infected groups represents the change in correlation strength, with positive values indicating stronger correlations in the *E. coli*-challenged group and negative values indicating weaker correlations. The correlation analysis of serum metabolites, between the control and *E. coli*-challenged broilers at 8 h, showed a consistent metabolite pair trend, but relationship strengths were generally stronger in the *E. coli*-challenged group (Figure S1) (Table S3F). The distinct change was observed between methylhistidine and adenine, increasing from R² = 0.72 of the control cohort to R² = 0.90 of the *E. coli* challenged group (ΔR² =+0.18; *p* = < 0.05). Further changes were observed as significant for deoxyguanosine and butyric acid. Isobutyric acid (R² = 0.77 to 0.91, ΔR²=+0.13; *p* = < 0.05). Indeed, nearly all metabolite pairs showed increased correlations in the infected cohort, except a slight decrease in the correlation of deoxyguanosine and adenine (ΔR²=−0.01; *p* = < 0.05). This data suggests that *E. coli* infection induces a more tightly regulated metabolic network, probably indicating coordinated host response to the bacterial challenge.

The correlation analysis at 24 h post-*E. coli* infection showed altered metabolite pair relationships as compared to the control group. These correlation changes primarily highlight the metabolic reprogramming of nucleoside, polyamine, and fatty acid pathways in *E. coli* challenged broilers (Figure S1) (Table S3G). N1-acetylspermidine/spermidine pair showed a ΔR² = 1.64 value with a strong shift of positive correlation in control birds (R² = 0.969) to negative correlation in *E. coli*-challenged broilers (R² = −0.67; *p* = < 0.05). This shift demonstrates the severe dysregulation of polyamine biosynthesis in *E. coli* challenged broilers. Additionally, N1-acetylspermidine showed significant losses of correlations with different nucleosides, such as deoxyinosine (ΔR² = 1.581; *p* = < 0.05), adenosine (ΔR² = 1.57; *p* = < 0.05), cytidine (ΔR² = 1.52; *p* = < 0.05), and adenine (ΔR² = 1.43; *p* = < 0.05) with all shifts from positive correlations in the control group to negative correlations in the *E. coli* infected group. All these changes pointed to the coordinated and systemic disruption of nucleoside-polyamine metabolic interactions as part of the host response to *E. coli* challenge.

Apart from nucleosides, N1-acetylspermidine showed a significant correlation shift with valeric acid/isovaleric acid (ΔR² = 1.38; *p* = < 0.05), implicating disruptions in polyamine and fatty acid metabolism. Conversely, the betaine/LysoPC.a.C18.1 pair presented an opposite shift from a negative correlation in the control group (R² = −0.46) to a positive correlation in the *E. coli*-challenged group (R² = 0.57, ΔR² = −1.03; *p* = < 0.05). This indicates a reorganization of phospholipid and one-carbon metabolism pathway in infected broilers.

These correlation results confirm that *E. coli* challenge induces a systemic metabolic network rewiring, specifically affecting polyamine biosynthesis, nucleoside metabolism, and phospholipid pathways. The key observation of consistent correlation losses centered around N1-acetylspermidine underscores it as a key hub in the metabolic response to *E. coli* infection in broilers.

## Network mapping of key metabolites in *E. coli* response

We have built R²-weighted correlation metabolite networks to identify the clusters of functionally coregulated metabolites in *E. coli*-challenged chickens at 8 and 24 h. At 8 h, the control group showed a coordinated metabolic correlation network comprising 8 metabolites with 12 interactions, compared to the challenged group, whereas the *E. coli*-infected group displayed a slightly reduced network of 7 metabolites with 11 interactions. Key metabolites like methylhistidine, deoxyguanosine, and butyric acid–isobutyric acid displayed increased centrality in the control group. However, orotic acid and N-acetyl-valine connectivity that was present in the control group was lost in the infected group, pointing out the metabolic network disruptions associated with early infection (Figure S2).

At 24 h, there was significant fragmentation in the metabolic network of *E. coli*-challenged broilers with 34 metabolites and 211 connections versus 38 metabolites and 652 connections in control broilers. Key metabolites such as N1-acetylspermidine, deoxyinosine, and adenosine flipped from strongly positive to negative correlations (Figure S2). Although N1-acetylspermidine remained highly connected, its interacting partners completely changed, and metabolites like oxalic acid and 3-hydroxybutyric acid lost all connectivity, indicating inactive pathways. Despite these changes, metabolites such as adenine and glycine remained highly connected, reflecting more subtle regulatory changes. The metabolite correlation networks revealed distinct changes in metabolic interactions between control and *E. coli*-infected broilers at 8- and 24-h post-infection.

## DISCUSSION

The early diagnosis and prediction of pathogenic infections caused by lethal bacterial infections such as *E. coli* is crucial in broiler chicken production, providing substantial benefits during the first week of their life. One of the core priorities of the broiler chicken industry is enhancing disease diagnosis capabilities to improve mitigation plans to control economically important diseases at the earliest convenience to improve profitability and food safety. *E. coli* septicemia is the most common pathogen associated with broiler chicken first-week mortality. Blood culture is currently the gold standard, but this technique requires more than 2 days for results. The specific biomarkers associated with early detection of pathogens are lacking. The present study aimed to fill this gap by exploring the metabolomics approach to identify pathogens such as *E. coli* within 24 h of exposure. This method will assist detection of infectious diseases before the onset of clinical signs, hence improving food security and animal welfare. Furthermore, as previously reported, mortality in this *E. coli* septicemia animal model, peaked at 2–3 days post- *E. coli* challenge, hence we wanted study metabolic changes at the early onset of septicemia^16^. The objectives of this study were to explore the metabolomics landscape of neonatal broiler chickens following *E. coli* septicemia and to identify potential biomarkers in the serum associated with *E. coli* septicemia. Our study employed targeted metabolomics technology to analyze peripheral blood metabolites in broiler chicks with sepsis at 8 and 24 h post-*E. coli* challenge.

Our results clearly demonstrated that as early as 8 h post-infection, metabolites such as adenine followed by N-acetyl-alanine, N-acetyl-isoleucine, N-acetyl-valine, and orotic acid linked to nucleotide and amino acid metabolism were downregulated. At 24 h, a distinct metabolic shift emerged with increasing hippuric acid while adenine further depleted and accompanied by decreases in N1-acetylspermidine, N-acetylputrescine, and a modest increase in picolinic acid in peripheral blood.

Adenine, C_5_H_5_N_5_ with molecular weight of 135.13 g/mol, is a purine nucleotide base, one of the four aromatic bases found in DNA and RNA^17^. Adenine operates at a molecular level, primarily through its role in the structure of nucleotides by carrying genetic information. Also, it is a component of adenosine and plays many important roles in different functions such as metabolic energy [adenosine triphosphate (ATP)], secondary messenger [cyclic adenosine monophosphate (cAMP)] and vasodilation (adenosine as a neurotransmitter)^18^. Adenine nucleotides play an important role in the transfer of energy for metabolic processes. Degradation of adenine can be a major source of purine bases formed during certain kinds of metabolic stress^19^. Adenine has been identified as a biomarker for diabetic kidney disease in humans and mice^20^. Also, it is required for the suppression of an immunological response. Adenosine levels in the extracellular space quickly increase in response to systemic inflammation or tissue damage. Plasma adenosine concentrations were observed to rise tenfold in septic shock patients^21,22^. This explains the downfold of adenine as it acts as a building block of adenosine.

A previous study reported a marked decreased in adenine levels in septicemic mice at 16–24 h post-infection^23^. Significantly, lower levels of adenine in collected blood (8 h: −59.5%, 24 h: −83.2%) can be primarily due to a significant drop in the production of ATP, the primary energy molecule that contains adenine as a key component. Our results align with findings in septic mice and humans, where systemic inflammation drives adenine consumption for nucleotide salvage pathways or ATP breakdown^24^. The adenine depletion is likely due to mitochondrial dysfunction impairing ATP synthesis^25^ and accelerated ATP catabolism in damaged tissues and immune cells, which release adenine and hypoxanthine for metabolic conversion, effectively draining circulating adenine reserves^26^. Normally, low adenine levels in the body are maintained by salvage pathways, which recycle it into nucleotides. The observed adenine depletion suggests that a pathological shift occurs where either it is used for clonal expansion of immune cells (lymphocytes) or the host’s salvage mechanisms are overwhelmed by metabolic stress. This phenomenon is consistent with reports in layer chickens infected with *Salmonella* Enteritidis, where serum like phosphocreatine, a critical energy reservoir for ATP regeneration, remained depleted for four days post-infection, indicating sustained disruption of nucleotide metabolism^27^. A modified adenine (N6-methyladenine) was identified as a useful metabolite for diagnosing up to 90% of urinary tract infections species in humans^28^.

*E. coli* is generally not an adenine auxotroph. Wild-type *E. coli* can synthesize adenine from simpler molecules and does not require an external supply of adenine for growth^29^. However, mutant strains of *E. coli* can be created that are auxotrophic for adenine, meaning they require an external source of adenine to survive. The adenine depletion observed in our study is likely due to massive host responses associated with proinflammatory responses and high energy demand linked with immune functions to eliminate *E. coli*. Some *E.coli* can obtain exogenous adenine (purine auxotrophy) as shown by coculture of *E. coli* with nucleotides in which *E.coli* showed rapid growth in media supplemented with adenine and derivatives like adenosine^30^.

Our metabolic pathway analysis revealed a decrease of N-acetyl-alanine (−50.2%), N-acetyl-isoleucine (−51.9%), N-acetyl-valine (−41.8%) in blood at 8 h post-*E. coli* infection. The metabolic spectrum of amino acids and amino acid metabolism changes dramatically during sepsis. As the disease progressed further with poor prognosis, the levels of the different amino acids gradually increased, decreased, or fluctuated over time^31^. In addition, the catabolic rates of patients with sepsis are significantly higher than their anabolic rates, the lower levels of alanine, isoleucine, and valine, have been shown to promote protein catabolism and reduce muscle protein synthesis^32^.

We observed elevated hippuric acid (+ 218.7%) in blood. It has been reported in human and mice studies, that elevated levels of uric acid in patients with sepsis are associated with an increased risk of acute kidney injury^33^. In addition, our analysis showed increased butyric acid/isobutyric acid (log estimate: 8.59, *p* = < 0.04) indicating early infection stage leads to changes in short-chain fatty acid metabolism and gut microbiome composition by increasing intestinal permeability, allowing bacterial toxins into the bloodstream and thus setting off systemic inflammation. Previously we reported upregulated butyric acid in the jejunum following *Clostridium perfringens* infected birds compared to non-infected healthy broilers^34^.

The projection of these metabolites on metabolic pathways suggested that the energy production pathways have changed. It is tempting to speculate that such changes in energy pathways may influence cellular and molecular recruitment. Our findings highlighted that the dynamics of metabolic pathways alter soon after pathogen exposure, even before the initiation of clinical signs. Clinical outcome was observed at 24 h post-infection where one bird from the challenged group died (3.3% mortality) while the rest of the birds in the group did not show any clinical signs. On necropsy, the dead bird had polyserositis (a combination of airsacculitis, pericarditis and perihepatitis). This emphasizes the importance of identifying a biomarker at the earliest possible time to initiate control strategies to reduce economic losses and improve animal welfare.

Our metabolomics data at 8 h and 24 h post-*E. coli* challenge indicated broilers experienced a biphasic host-response involving initial metabolic suppression then immune hyperactivation^35-37^. There is also substantial evidence that avian pathogenic *E. coli* (APEC) infection in chickens may lead to a cytokine surge like increased release of primary pro-inflammatory cytokines such as IL-1β, IL-6, and tumor necrosis factor (TNF)-α, which are hallmark mediators of sepsis^37^. At 8–12 h post-APEC infection, broilers demonstrated acute inflammation, such as edema, hyperemia, and heterophil infiltration in affected organs^36^. This immune hyperactivation manifests as an overwhelming inflammatory state that, while aimed at controlling the bacteria, can contribute to tissue damage and clinical symptoms of sepsis including fever, inflammation, and organ dysfunction. After this immune activity peak, it is plausible that chickens, like other animals, experience a downward shift in immune activity, leading to an immunosuppressive phase that is relevant to survivors of the acute stage. These survivors could be at potential risk of secondary bacterial infections. Our metabolite network showed shifting from coordinated regulation at 8 h to full disruption by 24 h, aligning with the cytokine storm, where metabolic instability, which could worsen clinical symptoms.

Biomarker-based diagnostic platforms utilize measurable indicators in biological samples to detect, diagnose, or monitor diseases. These platforms range from traditional laboratory tests to advanced technologies like microfluidics, wearable sensors, and AI-powered image analysis. Of these different platforms, colorimetric platforms for biomarker detection offer a versatile, affordable and accessible approach to disease diagnosis in the food-animal industry, particularly in resource-limited settings^38^. They utilize color changes to indicate the presence and concentration of specific biomarkers, enabling rapid, cost-effective, and portable diagnostics. Colorimetric platforms can detect biomarkers even at low concentrations, allowing for early disease diagnosis, which is crucial for effective treatment and improved disease management in the poultry industry. Colorimetric platforms would be advantageous under field conditions in the poultry industry using a blood sample as a rapid diagnostic material.

Based on our metabolomics data, it is clear that the progressive depletion of adenine as early as 8 h post *E. coli* infection, supports adenine as a potential metabolite biomarker of diagnosing *E. coli* septicemia in broiler chickens. However, to enhance diagnostic accuracy, it is advisable to implement a broader metabolite panel that captures unique metabolic signatures at both 8 h and 24 h post-infection. At the 8 h mark, increased concentrations of butyric acid/isobutyric acid and methylhistidine, alongside decreased levels of N-acetyl-isoleucine, N-acetyl-alanine, and orotic acid, may indicate early infection stages. By 24 h, upregulation of hippuric acid, N1-acetylspermidine, picolinic acid, and cholesteryl ester (22:2) suggests advanced-stage infection. The reproducibility of our results across two independent experiments underscores their reliability. It is important to validate this biomarker for its specificity and sensitivity in field conditions and assess its correlation with age and disease severity in broiler chickens under different management systems. Developing a rapid, reliable detection method for clinical use would be beneficial to the poultry industry. Implementing and validating a diagnostic panel that monitors these metabolites at the specified time points could enhance early detection and management of *E. coli* infections in poultry thereby improving flock health and productivity.

## Materials and methods

### Housing and maintenance of broiler chickens

All experimental procedures were performed in accordance with ARRIVE (Animal Research: Reporting of In Vivo Experiments) guidelines. The study was approved by the University of Saskatchewan Animal Research Ethics Board (protocol number 20070008). All methods were performed in accordance with the guidelines and regulations of the Canadian Council on Animal Care. Euthanasia was performed by cervical dislocation by well-trained personnel who are regularly monitored to ensure proficiency following the American Veterinary Medical Association guidelines for the euthanasia of animals. Mixed sex (male and female) Ross 308 broiler chicks were obtained from an in-house naive broiler breeder flock at the Animal Care Unit (ACU), Western College of Veterinary Medicine, University of Saskatchewan. Broiler chicks were randomly allocated into experimental groups in the animal isolation room at the ACU at the day of hatch. The housing and maintenance of broilers were done according to the recommendations by Aviagen Inc. In the ACU, the air exchange was controlled using HEPA filters for air exhaust, and the non-recirculated air is supplied at 15–20 air changes per h. The air pressure differentials and strict sanitation were maintained in this isolation facility. Broilers were raised at 32 °C for the first 7 days of life. Lighting (30–40 lx) was provided continuously until 2 days post-hatch, thereafter lux and duration were decreased until 10–20 lx and 7 h of darkness were achieved. *E. coli* infected birds and saline inoculated birds were kept in two separate rooms. Birds were fed *ad libitum* with a commercially available 20% raised without antibiotics broiler starter (Masterfeeds, Humbbolt, SK. Canada). Water was provided *ad libitum.*

### Culturing of *E. coli* and challenge

The challenge strain used was a field isolate of *E. coli* from a turkey showing septicemia. The *E*. *coli* isolate belonged to serotype O_2_, non-hemolytic, aerobactin producing, serum resistant, and has a K1 capsule^16^. The identity of the *E. coli* isolate was confirmed by matrix assisted laser desorption ionization-time of fight (MALDI-TOF) mass spectrometry in each experiment. First, the challenge bacteria were cultured on 5% Columbia sheep agar and incubated at 37 °C for 24 h. Then one colony was transferred to 100 mL of Luria broth (Difco LB broth, Miller, Becton Dickson and Company, USA) and incubated at 37 °C at 150 rpm for 18 h. After incubation, stationary phase *E. coli* was serially diluted in phosphate-buffered saline (PBS) to the desired challenge concentration of 1 × 10^5^ CFU/mL. Two experiments were conducted to assess changes in the metabolomic profile of broiler chickens at different time points following challenge with APEC.

Although *E. coli* septicemia in broiler chicks commonly occurs within the first week of life with high mortality, onset of *E. coli* infection could occur any day during the first week of life. We hypothesized that metabolic changes were similar in neonatal broiler chicks following challenge with *E. coli* irrespective of the day of exposure to *E. coli* hence, we challenge birds with *E. coli* at 3 and 5 days of age in two separate experiments.

## *Escherichia coli* challenge and sample collection at 8 h

A total of 60 broiler chicks (0 days of age) were randomly assigned into two groups (*n* = 30 birds/group). The number of birds per group was determined through statistical power analysis with power of 80% and significant level of 0.05 considering previous studies^16,39^. On day 5 post-hatch, one group of birds was subcutaneously inoculated with 1 × 10^5^ CFU/mL of *E. coli* in a volume of 250 µL per bird in the neck. The control group was inoculated with sterile saline (250 µL/bird) by the subcutaneous route in the neck. Following *E. coli* challenge, the broilers were monitored for clinical signs. Post 8 h following *E. coli* challenge, blood samples were collected from jugular vein from each bird with minimum handling and before euthanasia to minimize stress. Broilers from both the unchallenged control group and the *E. coli*-challenged group were examined for gross lesions on necropsy.

### *Escherichia coli* challenge and sample collection at 24 h

The second experiment followed the same protocol as experiment 1, with a modified challenge day and sampling point. Total of 60 broiler chicks were randomly assigned to two groups of *n* = 30/group at the day of hatch. One group was challenged with *E. coli* at 3 days of age, while the control group was inoculated with sterile saline (250 µL/bird) by subcutaneous route at neck. Blood samples were taken at 24 h post-infection.

### Separation of serum and Preparation of samples for metabolomic analysis

Immediately after blood collection from the control and *E. coli*-challenged groups, the samples were centrifuged at 1000 g for 10 min to separate the serum. Then, the serum was aliquoted into 1.5 mL microcentrifuge tubes, flash-frozen, and stored at −80 °C until further metabolomic analysis. All the samples were submitted for analysis using liquid chromatography-mass spectrometry (LCMS) to the Metabolomic Innovation Center (TMIC), in Alberta, Canada.

### TMIC mega metabolomics assay

TMIC Mega Metabolomics assay that uses a dual approach combining direct infusion (DI) mass spectrometry and reverse-phase liquid chromatography-tandem mass spectrometry (LC-MS/MS) (Applied Biosystems/MDS Analytical Technologies) instruments are used in this study. This assay could analyze up to 900 endogenous metabolites including amino acids, sugars, organic acids, nucleobases, vitamins, and different lipids, including sphingomyelins and triglycerides^17^. In this assay, isotope-labeled internal standards (ISTDs) at 1–10 µM concentration, along with chemical derivatization reagents like phenylisothiocyanate (PITC) for amino acids and 3-nitrophenylhydrazine (3-NPH) for organic acids, were added for boosting ionization and separation during mass spectrometry. Then, stock solutions of each analyte were prepared at a concentration range of 0.01-1 mM. All the above stock solutions were diluted to prepare the calibration standards (Cal1-Cal7) and quality control standards at low, medium, and high-0.05, 0.5, and 5 µM, respectively.

For the PITC derivatization step, all the samples were dried under nitrogen followed by 5% PITC treatment and then extracted by an injection of methanol: 5 mM ammonium acetate. For LC-MS/MS analysis, 50 µL from each extract was pipetted in a 96-well plate and mixed with 450 µL of LC/MS water. Chromatographic separation was performed on a reverse-phase C18 column (Waters Acquity UPLC BEH C18, 2.1 × 100 mm, 1.7 μm) using a binary solvent system consisting of water with 0.1% formic acid (solvent A) and acetonitrile with 0.1% formic acid (solvent B). The gradient started at 5% B, increased linearly to 95% B over 10 min, was held for 2 min, and then returned to initial conditions. Detection was performed using electrospray ionization (ESI) in both positive and negative modes, depending on metabolite class. Organic acids were derivatized with 250 mM of 3-NPH dissolved in 50% aqueous methanol, mixed for 2 h, and further diluted with water and butylated hydroxytoluene. For DI-MS/MS analysis, the remaining 10 µL of the extract was added with 490 µL of direct flow injection buffer for lipids, acylcarnitines and glucose/hexose identification. To ensure accuracy and precision, three quality control (QC) samples at low, medium, and high concentrations were checked in triplicate for reproducibility. Data normalization was done with internal standards, and for each metabolite LOD and limit of quantification (LOQ) were determined. Blank samples were used to check sample contamination and possible carryover effects. Analytical accuracy was validated by evaluating recoveries of spiked ISTDs in QC samples, including isotope-labeled amino acids, with recovery rates consistently between 85 and 115%, confirming reliable quantification across metabolite classes.

### Metabolomics data analysis

Metabolite concentration data (in µMs) from both experiment 1 and 2 were preprocessed to ensure reliability and comparability. Missing values below LOD were treated via imputation by replacing them with half the minimum detected concentration for each metabolite. Logarithmic transformation (log2) was applied to stabilize variance and normalize data distribution.

Initially, for the univariate analysis, student t-test was performed with limma package in R, for identifying metabolites that were differentially expressed between *E. coli*-challenged and the control group. For multiple comparisons, p-value adjustment was done with the FDR method, with the significance threshold set at FDR < 0.05.

In the multivariate analysis, we used both unsupervised and supervised methods. PCA analysis was performed using the ‘ggfortify’ in R package to visualize the overall structure of metabolomic data, unsupervised metabolic trends across experimental groups, and identification of outliers across experimental groups. Also, PLS-DA and OPLS-DA scores (VIP > 1) were applied to further investigate metabolic differences among the experimental groups. Unlike PCA, which focuses on overall variance, PLS-DA is a supervised method that reaches a maximum covariance between metabolite levels and groupings such as *E. coli*-challenged versus control, indicating which metabolites vary the most between groups.

Thereafter, for further validation of significant metabolites, meta-analysis approaches have been conducted through SAM and EBAM. Although both univariate and multivariate analyses identified some key metabolites, d-score, a modified t-statistic used in SAM was applied to correct for multiple testing to reduce false positives by permutation testing (*p* = < 0.05). EBAM improved further with the addition of Bayesian techniques yielding robust estimates of the posterior probability of metabolites being truly significant, along with z-scores and *p*-values (*p* = < 0.05). These approaches ascertain that the metabolic changes identified were not through random variation but rather a true biological effect of the *E. coli* infection in both experiments.

To further consolidate findings, a Venn diagram was constructed to compare metabolite data from univariate, chemometric, and meta-analysis approaches. This identified robust metabolite consistently detected by multiple methods. The shared and unique metabolites were compared by using another Venn diagram.

Then, the core metabolite sets across both experimental groups were subjected to pathway enrichment analysis (Human Metabolome Database and Kyoto Encyclopedia of Genes and Genomes) using MetaboAnalyst with *Gallus gallus* as a reference organism. Significantly enriched pathways were identified by comparing query metabolites versus totally known metabolites in that pathway (*p* = < 0.05). The metabolites identified in this stage were subsequently used in logistic regression analysis. Here, we calculated the *p*-value and effect size for metabolites across both datasets to assess their importance in distinguishing both the experimental groups. A CS was derived using the formula, CS (effect size × -log10(P)), to combine the magnitude of the metabolite effect change with its statistical reliability. Metabolites with high CS score were identified to be metabolite biomarkers for *E. coli* infection.

Construction of correlation maps and network analysis of significant metabolites from LR findings were conducted to analyze the strength of associations between metabolites and also the hub metabolites involved in *E. coli* infection. For correlation analysis, where R² value ranges from 0 to 1, where a value closer to 1 indicates that a large proportion of the variance is explained by the model, suggesting a good fit.

The statistical analysis and visualization was primarily done with R software tools, including lemma^18^ggplot2, ggpubr, Enhanced Volcano, and pheatmap. Pathway analysis and network visualization was done using XCMS tool, MetaboAnalyst, and Cytoscape tools. A *p-*value of less than 0.05 (FDR < 0.05) was considered statistically significant for most statistical tests.

## Supplementary Information

Below is the link to the electronic supplementary material.

Supplementary Material 1

Supplementary Material 2

## Data Availability

All metabolomics data supporting the findings of this study are provided within the manuscript and its supplementary information files, including raw and processed excel tables labeled Table S1–S3. In addition, the original raw data have been deposited in Zenodo and are publicly available at https://zenodo.org/records/16138868.
