# Supplementary material for: Temporal metabolomic fingerprinting identifies adenine as a novel biomarker for early detection of Escherichia coli infection in broiler chickens
Source: Sci Rep. 2025 Aug 27;15:31580. doi: 10.1038/s41598-025-16873-x (PMC12391293; doi:10.1038/s41598-025-16873-x)
Supplement: Supplementary file 2 — Supplementary Material 2 [file 41598_2025_16873_MOESM2_ESM.pdf]

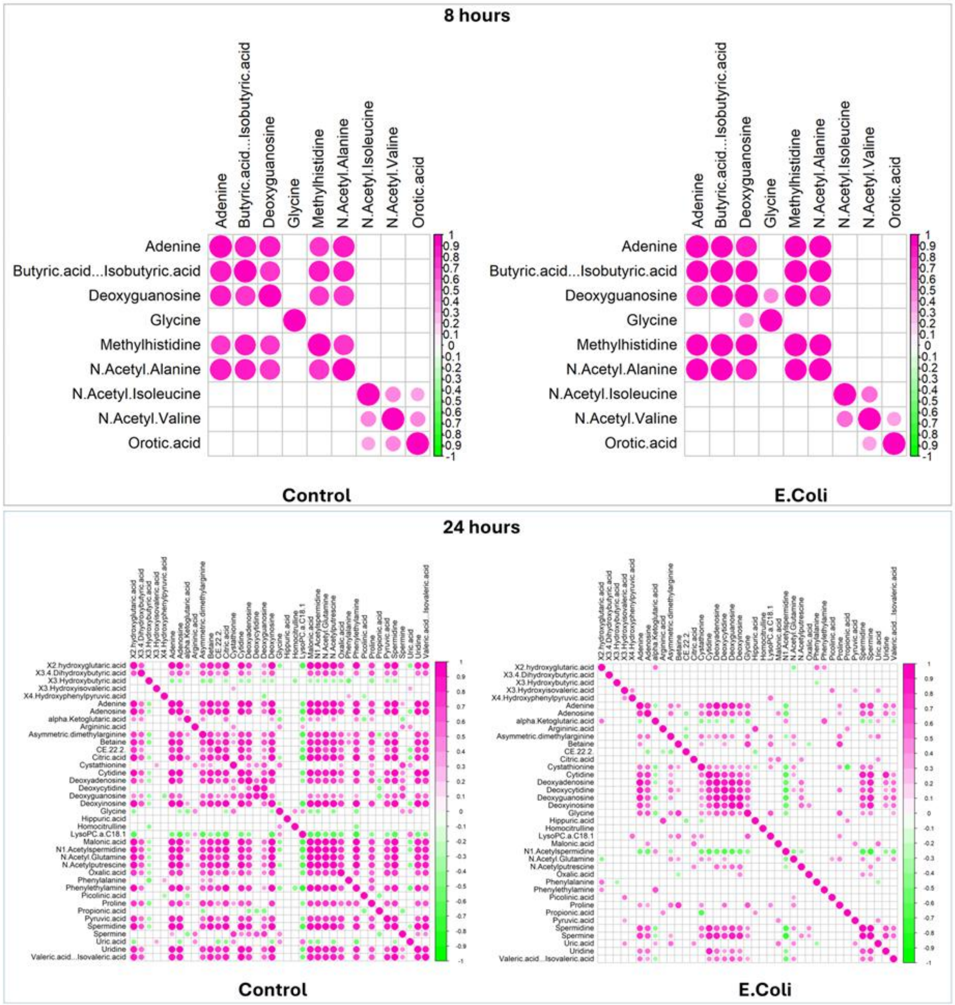

Figure S1: Correlation Matrices of Serum Metabolites in Control and *E. coli* Challenged Chickens: The figure shows correlation matrices for serum metabolites in control (left) and *E. coli*-challenged (right) groups at 8 hours (top) and 24 hours (bottom) post-infection. Dot size and color represent Pearson correlation strength and direction: magenta for positive and green for negative correlations, with larger dots indicating stronger relationships. The matrices highlight metabolic interaction changes during early (8 hours) and late (24 hours) *E. coli* infection.

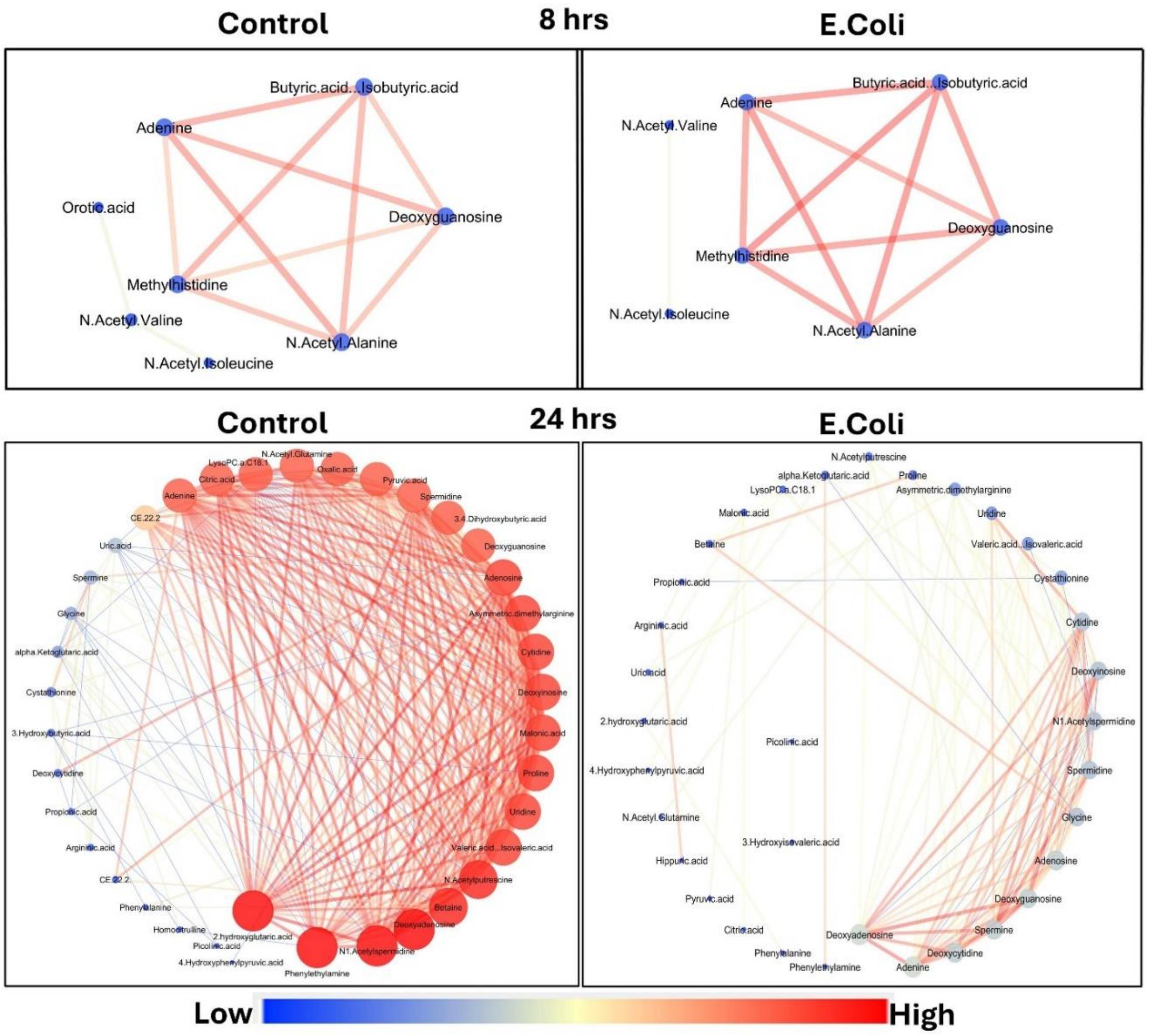

Figure S2: Metabolite correlation networks for control (left) and *E. coli*-infected (right) chickens at 8- and 24-hour post-infection. Nodes represent metabolites, and edges indicate Pearson correlations. Line thickness and color (red for high positive, blue for low) reflect correlation strength (R^2 values), while node size indicates metabolite centrality. At 8 hours post-infection, *E. coli*-challenged chickens showed stronger and more coordinated metabolic interactions compared to controls, while at 24 hours, significant disruptions in polyamine, nucleoside, and fatty acid pathways emerged, indicating systemic metabolic rewiring in response to infection.
